# Suzetrigine, a Na_V_1.8 Inhibitor as a Novel Approach for Pain Therapy—A Medicinal and Chemical Drug Profile

**DOI:** 10.3390/molecules31020358

**Published:** 2026-01-20

**Authors:** Rawan M. Medhat, Omnia A. Kotb, Daniel Baecker

**Affiliations:** 1 Department of Pharmaceutical Chemistry, Faculty of Pharmacy, Pharos University in Alexandria, Alexandria 21648, Egypt; 2 Department of Clinical Pharmacy and Pharmacy Practice, Faculty of Pharmacy, Pharos University in Alexandria, Alexandria 21648, Egypt; 3 Department of Pharmaceutical and Medicinal Chemistry, Institute of Pharmacy, Freie Universität Berlin, Königin-Luise-Straße 2+4, 14195 Berlin, Germany

**Keywords:** suzetrigine, Na_V_1.8, sodium channel, acute pain, non-opioid analgesic

## Abstract

Suzetrigine was approved by the US American Food and Drug Administration in 2025 as the first oral, non-opioid, selective inhibitor of Na_V_1.8 sodium channel for the treatment of acute pain. Therefore, it represents a groundbreaking advancement in pain management. This review aims to provide an overview of the milestones in the medicinal-chemical development of Na_V_1.8 inhibitors, eventually leading to suzetrigine. The multi-step synthesis route of suzetrigine is presented. Taking structural features into account, insights are provided into what plays a role for the inhibition of the Na_V_1.8 channel. In addition, pharmacodynamic and pharmacokinetic aspects of the new drug, such as bioavailability, metabolism, and interaction with CYP450 enzymes, are discussed. A summary based on a large number of clinical trials demonstrating remarkable efficacy completes this comprehensive drug profile of suzetrigine, while also addressing limitations of the clinical trials and suggesting future perspectives.

## 1. Introduction

Acute pain, especially of moderate-to-severe intensity, is a common clinical condition that leads to considerable suffering. Thus, it has a significant impact on productivity and quality of life [1,2,3,4,5,6]. A multimodal approach is currently the recommended pain management strategy. This involves combining medications with different mechanisms of action to effectively control pain. Among these agents are acetaminophen, non-steroidal anti-inflammatory drugs (NSAIDs), opioids, *N*-methyl-D-aspartate receptor antagonists, and local anesthetics [7,8,9].

Opioids have been considered one of the most powerful analgesics available for treating moderate-to-severe acute pain for decades, particularly arising from surgery, injury, or inflammation. Despite their significant potency, the activity in the central nervous system (CNS) and long-term usage are associated with serious limitations, including tolerance and a high risk of addiction. This contributes to raising the burden of the current opioid epidemic [4,7,10,11]. Other classes of analgesics can cause a wide range of adverse effects, such as hepatotoxicity, renal impairment, gastrointestinal toxicity, and cardiotoxicity. As a result, there is an urgent need to develop a new class of non-opioid analgesics that provide safe and effective pain relief with a non-addictive profile [4,5,6,12,13,14,15].

Targeting voltage-gated sodium channels, particularly the subtype Na_V_1.8, is one of the novel pharmacological approaches for managing acute pain, due to its pivotal role in transmitting pain signals in the peripheral pain-sensing neurons (nociceptors) [1,4,6,16,17,18,19,20,21].

On 30 January 2025, the US American Food and Drug Administration (FDA) approved suzetrigine, developed and eventually marketed by Vertex Pharmaceuticals Inc. under the trade name JOURNAVX™, as the first oral, non-opioid, selective inhibitor of the Na_V_1.8 sodium channel for the treatment of moderate-to-severe acute pain [16,22,23,24,25,26]. This review aims at providing a comprehensive profile of suzetrigine, summarizing the history of its development, the chemical synthesis, clinical trials, pharmacodynamics, pharmacokinetics, and metabolism.

## 2. History of Na_V_1.8 Inhibitor Development

Although the Na_V_1.8 sodium channel is considered a promising target for peripheral pain management, the development of selective blockers remains challenging. This results from the high-degree of sequence similarity among the Na_V_ channel subtypes [5,27,28,29,30]. Researchers at several pharmaceutical companies, including Abbott, Pfizer, and Vertex, have made extensive efforts to design a prototype selective Na_V_1.8 inhibitor [20,29,31].

**Figure 1:**
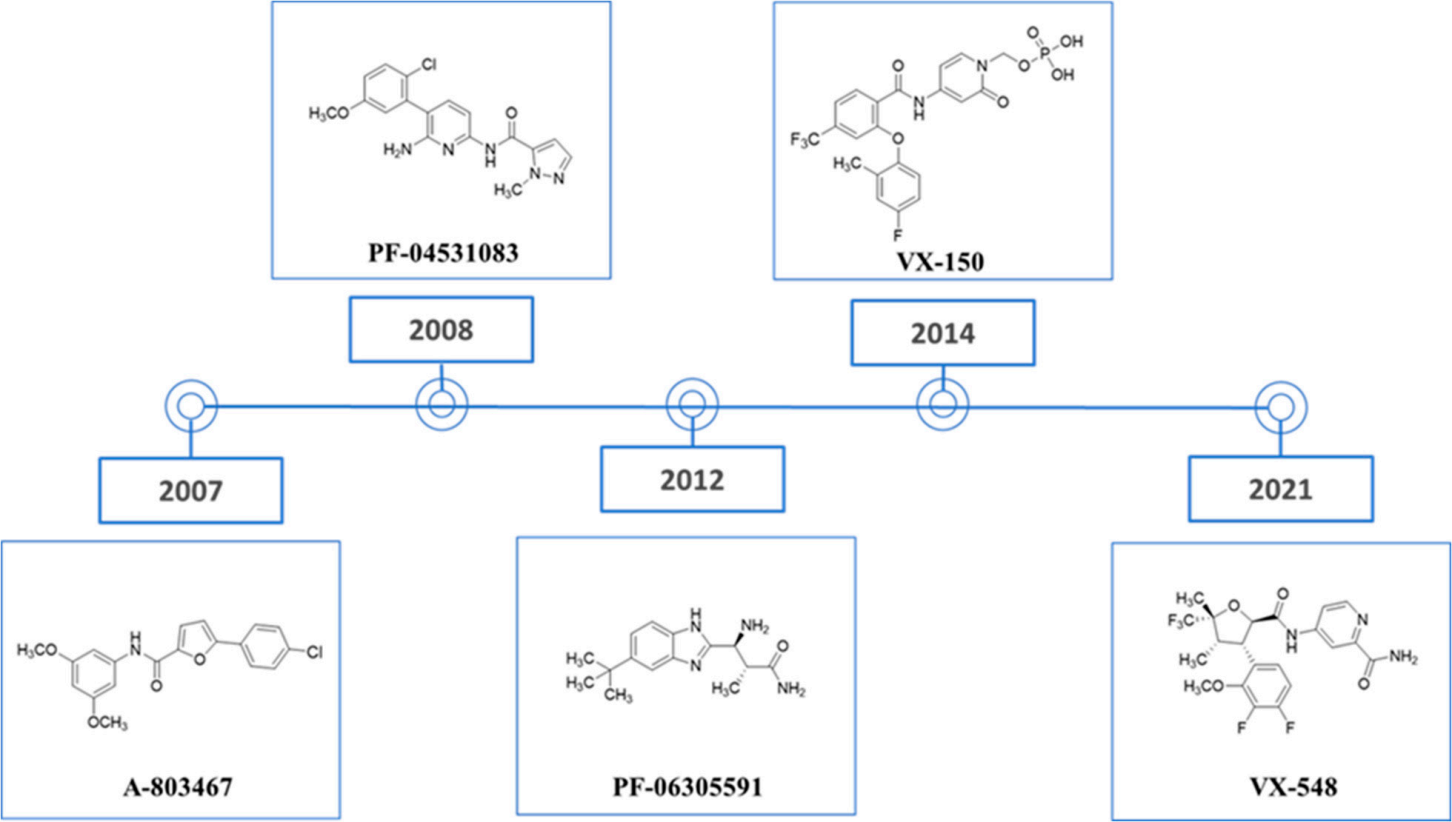
Notable milestones in the development of suzetrigine (VX-548) for the treatment of acute pain.

**Figure 2:**
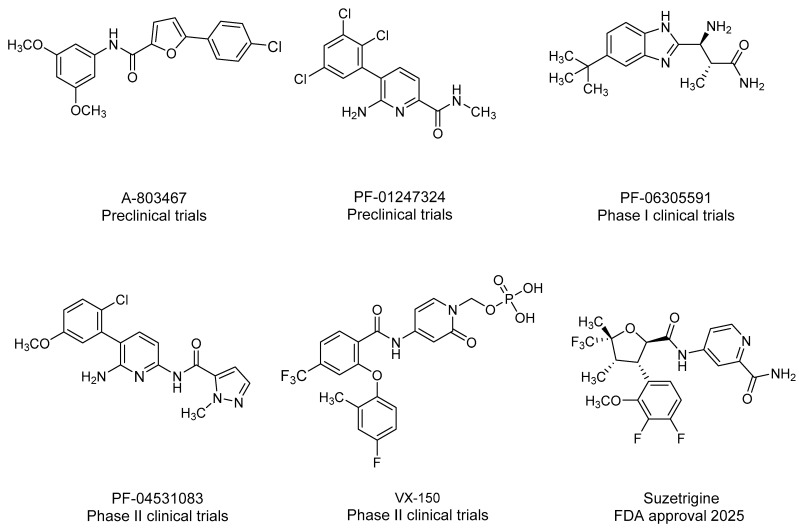
Chemical structures of selected Na_V_1.8 inhibitors.

A-803467 (see Figure 1) was one of the first compounds developed by Abbott Laboratories in 2007 as a selective Na_V_1.8 inhibitor. This compound demonstrated selectivity and 300–1000-fold greater potency for Na_V_1.8 compared to other Na_V_ channel subtypes in several preclinical trials [9]. This demonstrated the first evidence for the significant role of Na_V_1.8 in reducing inflammatory and neuropathic pain. However, it failed to proceed to clinical trials due to poor pharmacokinetics and limited efficacy in reducing other types of pain, including formalin-induced pain, postoperative pain, and acute thermal pain [7,9,27,32,33,34]. Subsequently, Pfizer reported the discovery of several compounds as selective Na_V_1.8 blockers for the treatment of pain, including PF-01247324, PF-04531083, and PF-06305591 (see Figure 1 and Figure 2).

PF-01247324 (see Figure 2) was designed based on the chemical structure of lamotrigine (3,5-diamino-6-(2,3-dichlorophenyl)-1,2,4-triazine). Lamotrigine serves as a weak, non-selective Na_V_ channel blocker used in the treatment of epilepsy and bipolar disorders [35]. The compound PF-01247324 showed promising results in preclinical trials using rat models for inflammatory and neuropathic pain. Despite its high potency and more than 50-fold selectivity for Na_V_1.8 over other Na_V_ channels, it did not advance to clinical trials [5,27,34,36].

In 2008, Pfizer disclosed PF-04531083, another compound structurally similar to lamotrigine, which exhibited high potency (IC_50_ < 200 nM) and limited cardiotoxicity, as it revealed no interaction with the human ether-a-go-go-related gene (hERG) potassium channel [5,37]. This drug afforded promising results in reducing neuropathic pain in rodents, with efficacy similar to that of pregabalin, i.e., (*S*)-3-(aminomethyl)-5-methylhexanoic acid, making it a candidate for clinical evaluation [5,27,34]. Several phase I clinical studies demonstrated a good safety profile and oral bioavailability [5,38,39,40]. However, a phase II clinical trial assessing the efficacy of post-surgical dental pain was terminated due to insufficient outcomes in pain reduction within 6 h [5,41].

By the end of 2012, Pfizer reported on PF-06305591, a benzimidazole derivative, as another selective Na_V_1.8 blocker for pain management. Although the results of its clinical trials have not yet been posted [42,43,44,45,46], some researchers considered it a back-up candidate for PF-04531083 [5,33].

In 2014, Vertex Pharmaceuticals introduced the first generation of selective Na_V_1.8 blockers with >400-fold greater potency compared to other Na_V_ subtypes [27,47]. This compound, a 2-pyridone amide derivative, was named VX-150 [48,49,50]. The promising results in phase I clinical trials for managing cold pressor and heat pain [7,47] enabled VX-150 to progress to a series of phase II clinical trials (NCT03304522). The latter involved patients with neuropathic and postoperative acute pain [27,51]. The first two clinical studies evaluated the efficacy and safety in patients with moderate-to-severe knee osteoarthritis [5,52] and small fiber neuropathy [51]. No serious adverse effects were reported, while the most common side effect was headache [53]. These studies were followed by two clinical trials in patients with acute surgical pain after bunionectomy, and the initial outcomes were promising [54]. However, further development was discontinued due to inadequate efficacy, as high doses were required to provide moderate pain relief, and also unwanted side effects, such as headache, were observed [7,27,51].

Finally, Vertex succeeded in overcoming the challenges faced in the previously reported Na_V_1.8 inhibitors and achieved a significant breakthrough in 2021 by developing VX-548 (suzetrigine), a second-generation Na_V_1.8 blocker. This compound is a highly potent inhibitor (IC_50_ = 0.7 nM), with 31,000-fold selectivity over other Na_V_ channels [7,27]. The remarkable efficacy was demonstrated in multiple clinical trials, including a phase III study (NCT05661734) [55] that showed broad-spectrum pain relief, especially in surgical pain after bunionectomy (NCT05553366) [56] and abdominoplasty (NCT05558410) [57,58]. Moreover, VX-548 also yielded promising results in phase II clinical trials for diabetic peripheral neuropathy (DPN) (NCT05660538) [59] and lumbosacral radiculopathy (LSR) (NCT06176196) [60]. This discovery resulted in the approval of VX-548 by the FDA in 2025 as the first non-opioid analgesic for the management of acute pain [1,27].

## 3. Synthesis

Structurally, suzetrigine (4-[(2*R*,3*S*,4*S*,5*R*)-3-(3,4-difluoro-2-methoxyphenyl)-4,5-dimethyl-5-(trifluoromethyl)oxolane-2-amido)pyridine-2-carboxamide, C_21_H_20_F_5_N_3_O_4_, M_r_ = 473.4) is a pyridine derivative. It has four stereogenic centers, all of which are located on the tetrahydrofuran (oxolane) core. In addition, the chemical structure of suzetrigine bears five fluorine substituents, arising from one trifluoromethyl substituent and one ortho-substituted difluorophenyl residue. The introduction of fluorine is of particular interest in medicinal chemistry [61,62], for example, to increase the metabolic stability of a drug.

**Scheme 1:**
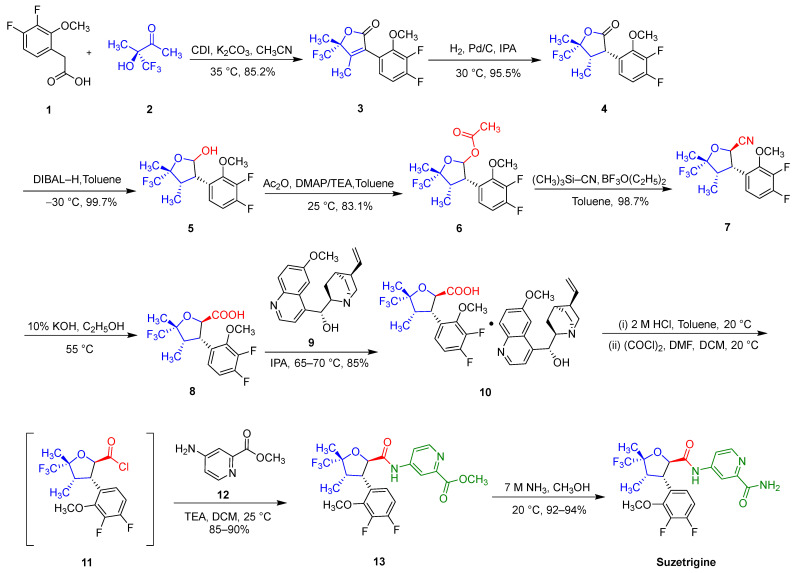
Chemical synthesis of suzetrigine.

The synthesis of suzetrigine, developed by Vertex Pharmaceuticals, is outlined in Scheme 1 [63]. The synthetic pathway started with the reaction of the twice fluorinated phenylacetic acid derivative **1** and 1,1′-carbonyldiimidazole (CDI) as a coupling agent in anhydrous acetonitrile to form the *N*-acyl imidazolide intermediate. The latter was then subjected to nucleophilic addition by the thrice fluorinated α-hydroxy ketone **2**, followed by lactonization to afford **3** in 85.2% yield. Hydrogenation of **3**, catalyzed by 5% palladium on carbon in isopropyl alcohol (IPA), reduced the double bond, yielding **4** in 95.5% yield. The carbonyl group of the lactone in **4** was reduced using diisobutylaluminium hydride (DIBAL-H) in anhydrous toluene, affording the hemiacetal moiety of **5** almost quantitatively. Compound **5** underwent esterification with acetic anhydride (Ac_2_O) in the presence of 4-dimethylaminopyridine (DMAP)/triethylamine (TEA) as a catalyst in anhydrous toluene at room temperature to obtain the acetate ester **6** (yield: 83.1%). The ester **6** was treated with trimethylsilyl cyanide in the presence of boron trifluoride etherate as a Lewis acid in anhydrous toluene, affording **7** also almost quantitatively. Subsequent KOH-mediated hydrolysis of the nitrile group in ethanol yielded the carboxylic acid **8**, which was subjected to chiral resolution using quinine **9** in IPA, producing the corresponding quinine salt **10** in 85% yield. The quinine salt **10** was first treated with 2 M aqueous hydrochloric acid in toluene to liberate the free acid **8**, which was then extracted. The resulting acid **8** was dissolved in dichloromethane (DCM) and treated with oxalyl chloride in the presence of *N,N*-dimethylformamide (DMF), generating the acid chloride **11**. Subsequently, **11** reacted with methyl 4-aminopicolinate **12** in the presence of TEA in DCM to obtain **13** in 85–90% yield. Finally, the desired suzetrigine was obtained by reacting **13** with ammonia solution in methanol with a yield of 94% for transforming the ester to the respective amide.

## 4. Structural Insights into Na_V_1.8 Channel Inhibition

Na_V_1.8 comprises one alpha-subunit, which is essential for channel activity, and two auxiliary beta-subunits that help in membrane localization and channel modulation [64,65,66,67,68,69]. The alpha-subunit, containing the binding sites for small molecule inhibitors, is composed of four domains (DI-DIV) connected by intracellular loops, while each domain consists of six transmembrane segments (S1–S6) [20,70,71,72,73]. The S1–S4 segments constitute the voltage-sensing domain (VSD), whereas S5 and S6 form the central ion-conducting pore domain (PD) and are linked by a pore loop (P-loop) [64,70,72,74,75,76].

Although the crystal structure of suzetrigine in complex with Na_V_1.8 has not yet been reported and remains challenging to determine due to the multiple conformational changes exhibited by Na_V_ channels [31,77,78], several cryogenic electron microscopy, mutagenesis, and electrophysiological studies have provided remarkable insights into the precise binding site of Na_V_1.8 inhibitors and their mechanism of action [6,27,79,80]. These studies revealed that suzetrigine binds to the extracellular loop of the S3–S4 region in the second voltage-sensing domain of Na_V_1.8 (VSD2), distant from the pore domain, indicating an allosteric channel inhibition [6,50,79,81].

**Figure 3:**
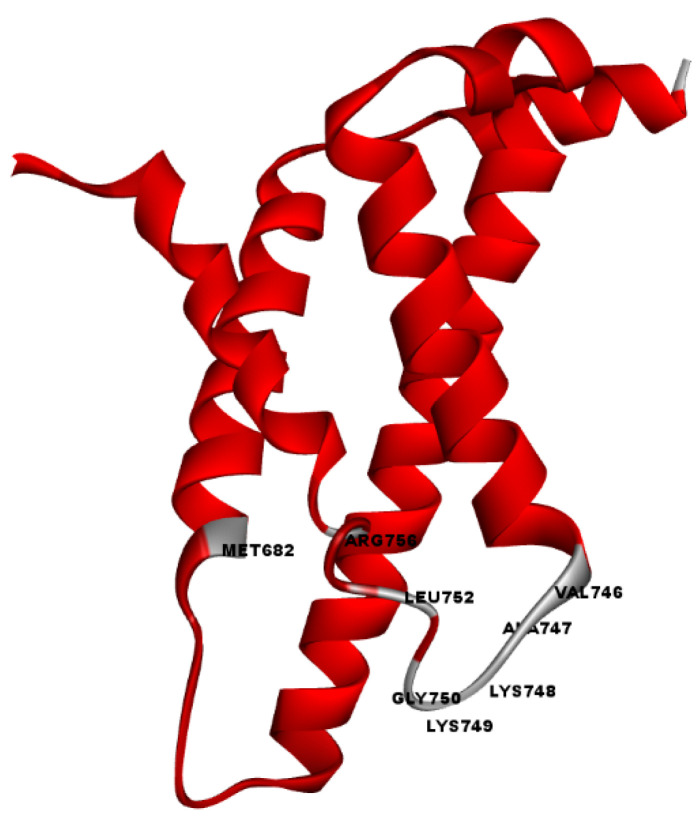
The predicted binding site of LTGO-33 in the extracellular cavity of VSD2 in Na_V_1.8, showing key interacting residues (PDB ID: 7WE4). Visualization was performed using Discovery Studio Visualizer 24.1.0.23298 (Dassault Systèmes BIOVIA, San Diego, CA, USA).

In 2024, Gilchrist et al. [77] reported on critical residues within the S3–S4 region of VSD2 of Na_V_1.8 that are essential for selectivity, including Val746, Lys748, Lys749, and Gly750 (see Figure 3). The study was conducted on LTGO-33 ((*R*)-2-(4-fluoro-2-methylphenoxy)-*N*-(3-(methyl sulfonimidoyl)phenyl)-5-(trifluoromethyl)pyridine-3-carboxamide), an orally active Na_V_1.8 inhibitor in preclinical trials developed by Latigo Biotherapeutics. This compound exhibited high potency and selectivity and turned out to bind at the same VSD2 site as suzetrigine [6,27,77,80,82,83].

To date, research on the structure–activity relationship of suzetrigine is still ongoing. However, several structural features are frequently observed among previously reported Na_V_1.8 inhibitors, including aryl-linked amides, the presence of a basic nitrogen, and the incorporation of fluorine atoms. The latter contribute to the binding to hydrophobic caves and increase stability against metabolic degradation [80]. These common features play an important role in enhancing potency, improving selectivity, and modulating physicochemical properties of Na_V_1.8 inhibitors [27,61,80,84,85,86,87].

## 5. Pharmacology

### 5.1. Mechanism of Action

Suzetrigine selectively inhibits the Na_V_1.8 sodium channel, a tetrodotoxin-resistant voltage-gated sodium channel encoded by the sodium voltage-gated channel alpha-subunit 10 (SCN10A) gene. The sodium channel Na_V_1.8 is highly expressed in peripheral pain-sensing neurons and is a key contributor to the initiation and transmission of nociceptive pain signals [4,6,9,20,24,27,88,89]. These characteristics make Na_V_1.8 a promising therapeutic target for a wide range of pain conditions, including acute, inflammatory, and neuropathic pain. Notably, neuropathic pain arises from a lesion or disease affecting the somatosensory nervous system, leading to persistent pain signaling [90,91]. Na_V_1.8 knockout studies in mice models have demonstrated reduced responses to acute noxious stimuli, as well as diminished inflammatory hyperalgesia, whereas these models fail to show a notable change in neuropathic pain responses [92]. However, human genetic studies have shown that gain-of-function mutations in Na_V_1.8 are associated with increased peripheral neuropathic pain responses [93,94]. Na_V_1.8 is also characterized by activation at more depolarized voltages compared to other sodium channels, along with slow inactivation, enabling it to maintain the action potential during repetitive firing. Thereby, its selective inhibition disrupts the transmission of pain signals from peripheral sensory nerves to the CNS [4,6,16,18,21,95,96].

Unlike non-selective sodium channel blockers, which block the sodium influx through the pore region of Na_V_ channels [97,98,99,100], suzetrigine binds to a unique allosteric site on the second voltage-sensing domain (VSD2) of Na_V_1.8, stabilizing the closed state of the channel and preventing sodium permeation in the peripheral nerves [6,24,101]. Recent studies showed that the affinity of suzetrigine for Na_V_1.8 channels can be reduced in the activated state, while the affinity remains strong in the resting state across a wide range of action potentials. This rare phenomenon is known as the reverse use-dependence state [6,22,79,82,97,98,99].

Therefore, suzetrigine is considered a promising drug candidate, as it provides peripheral pain relief with evidence-based efficacy and selectivity for Na_V_1.8 over other voltage-gated sodium channel subtypes [22] (Na_V_1.1 [22,102], Na_V_1.7 [22,103], Na_V_1.9 [22,104,105]). Furthermore, it displays an allosteric modulation distinct from that of local anesthetics, and it also demonstrates a favorable safety profile with no evidence of addictive potentials, distinguishing it from the abuse liability associated with opioids [4,6,7,106].

### 5.2. Pharmacodynamics

Suzetrigine is a highly potent Na_V_1.8 inhibitor (IC_50_ = 0.7 nM) with approximately 31,000-fold selectivity over other Na_V_ channels [7,27,94]. These findings were based on in vitro studies using cells expressing Na_V_1.1–Na_V_1.7 and Na_V_1.9 channels, including human embryonic kidney (HEK), Chinese hamster ovary (CHO), or rodent neuroblastoma fusion (ND7/23) cell lines. Suzetrigine also demonstrated high potency in neurons isolated from human dorsal root ganglion neurons, with an IC_50_ value of 0.68 ± 0.16 nM [1,6].

Comprehensive non-clinical studies were conducted to assess the safety and addictive potential of suzetrigine. Among these, the repeat-dose toxicity studies used concentrations exceeding the therapeutic dose in rats and monkeys. The results revealed that suzetrigine was not associated with effects on the CNS related to dependence or abuse [6]. Another CNS safety study reported that suzetrigine induced no significant behavioral changes, including stimulation or sedation in monkeys [6,81]. Cardiovascular safety assessments were also performed and revealed that suzetrigine produced no observable adverse cardiovascular effects, which was confirmed by the absence of changes in blood pressure and electrocardiogram [6,81,107].

Furthermore, several clinical trials have provided supportive evidence regarding the efficacy of suzetrigine and safety in the management of acute pain (see below).

### 5.3. Pharmacokinetics and Metabolism

The approved dose regimen for the application of suzetrigine comprises a 100 mg single loading dose and a maintenance dose of 50 mg every 12 h [16,108]. The administration of the loading dose with meals of varying calorie and fat content (high, moderate, or low) has shown to reduce the initial drug concentration. This effect was not observed when the dose was taken on an empty stomach. However, the absorption of the maintenance doses does not appear to be affected by food intake. Accordingly, it is recommended to take the initial dose on an empty stomach (2 h before or 1 h after drug administration), while the following doses can be taken independently of meals [16,108]. The primary pathway for the metabolism of suzetrigine is mediated by the cytochrome P450-dependent enzyme CYP3A. Suzetrigine is largely eliminated in both feces and urine in approximately equal proportions, and has a mean clearance of 13.9 L/h [107,109].

**Figure 4:**
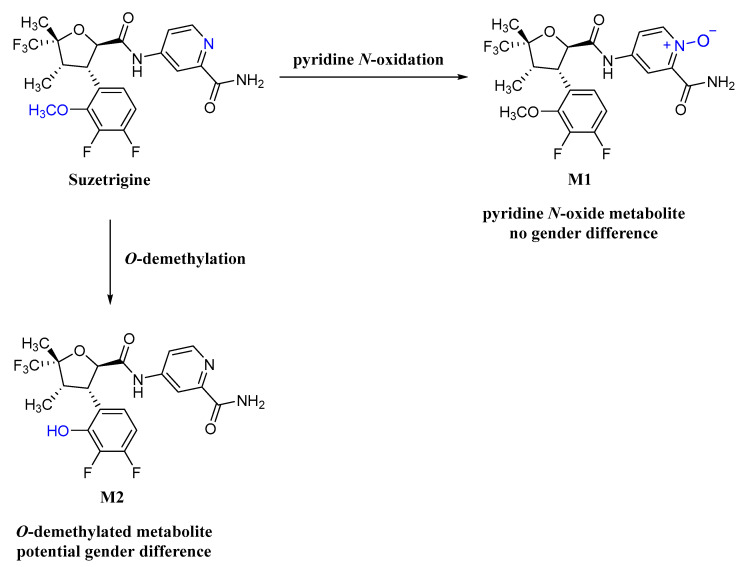
Metabolic biotransformation of suzetrigine in male and female rat hepatocytes [109,110].

The metabolites of suzetrigine detected in human hepatocytes are the pyridine *N*-oxide (M1) and the *O*-demethylated (M2) derivative (see Figure 4). The M1 metabolite was reported as the major active metabolite [107,109].

An in vitro study using male and female rat hepatocytes demonstrated a significant gender difference in the hepatic metabolism of suzetrigine. The results revealed that clearance was seven times faster in male rat hepatocytes than in females. In addition, the amount of the *O*-demethylated (M2) metabolite was ten times higher in male rats than that in females, whereas the amount of the pyridine *N*-oxide (M1) metabolite was the same in both genders, indicating that the gender difference in the metabolism of suzetrigine was related to the formation of the *O*-demethylated (M2) metabolite rather than the pyridine *N*-oxide (M1) metabolite [107,109,110]. Another in vitro study using human male and female hepatocytes showed no significant gender difference in hepatic metabolism of suzetrigine. The results revealed that the metabolism of suzetrigine in humans was similar to that observed in female rats [107,109,110] (see Figure 4).

The absolute bioavailability of suzetrigine has not been clearly addressed. However, its time to peak concentration (T_max_) has been reported to range from 1.5 to 5 h, with a mean of approximately 3 h, while its active metabolite (M1) exhibits a T_max_ ranging from 4 to 48 h, with an average of 10 h. Following administration of the initial dose, T_max_ is influenced by food intake, delaying T_max_ to 5 h for suzetrigine and 24 h for its metabolite (M1) [16,107,108]. Suzetrigine exhibits a moderate area under the curve (AUC) (11.5 μg·h/mL), whereas its maximum plasma concentration (C_max_) is relatively low (0.62 μg/mL) [73].

Both suzetrigine and its dominant active metabolite (M1) are highly protein bound, which means that any condition causing changes in the albumin level would need dose adjustment accordingly [22]. The plasma protein binding is approximately 99% for suzetrigine and 96% for its metabolite (M1). Suzetrigine has a large apparent volume of distribution (495 L), which contributes to the moderate to long half-life observed for both suzetrigine (approximately 24 h) and its active metabolite (approximately 33 h) [107]. The metabolite M1 has a longer effective half-life (33.0 h) and higher steady-state exposure when compared with the main drug (23.6 h). However, during an in vitro electrophysiology assay in human dorsal root ganglion neurons, M1 was a less potent inhibitor of Na_V_1.8 (about 3.7-fold weaker compared to suzetrigine) [16].

Being a CYP450-sensitize drug, different dose modifications according to patients’ pharmacogenomics, liver function, and polypharmacy become relevant. Although in mild hepatic impairment (Child-Pugh score A), no clinically significant changes occurred. Moderate impairment (Child-Pugh score B) caused the parent drug steady-state AUC during 12 h AUC_0–12 h_ to increase ~1.5-fold and C_max_ raised ~1.3-fold. The metabolite M1 exposures were enhanced ~1.2-fold. Thus, it is recommended to make a dose reduction in moderate impairment and to avoid its use in severe hepatic impairment (Child-Pugh score C) [16,108].

Drug–drug interactions are highly suspected when taken with other drugs acting as CYP3A inhibitors (e.g., reported with the antifungals itraconazole and fluconazole) or CYP3A4 inducers (e.g., reported with the antibacterial rifampicin and the antiviral efavirenz). Suzetrigine also works as a CYP450 enzyme inducer on CYP3A and to a lesser extent on CYP2B6 and CYP2C8/9/19. It was reported to reduce the AUC of the benzodiazepine midazolam by ~48% (a sensitive CYP3A probe), making dose modification necessary when co-administered. However, more studies are needed for detailed information on the extent of interaction [109].

There are some trials that were already completed, but their results are still not published. The studies NCT06336096 and NCT05851157 aimed at investigating the impact of food on the pharmacokinetics of suzetrigine in healthy participants [111,112]. The trial NCT06820307 had the goal to assess the effect of suzetrigine on the pharmacokinetics of oral contraceptives in healthy female participants [113]. The clinical trial NCT05818852 was designed to evaluate the effects of clinical and high-clinical exposures of suzetrigine and its metabolite M1 on QT interval corrected by Fridericia’s formula (QTcF), in addition to assessing the safety, tolerability, pharmacokinetics, and pharmacodynamics [114], which were also evaluated in two additional studies (NCT05704556 and NCT05560464) in participants with moderate or severe renal impairment and mild or moderate hepatic impairment [115,116].

Drug interaction trials (NCT05635110 and NCT05541471) have been performed to evaluate the pharmacokinetics and the safety of suzetrigine and its metabolite M1 in the absence and presence of the proton pump inhibitor omeprazole or the antibiotic rifampicin, in healthy participants, and the pharmacokinetics of the benzodiazepine midazolam and the cardiac glycoside digoxin in the absence and presence of suzetrigine [117,118].

## 6. Clinical Efficacy

### 6.1. Published Clinical Trials

There are several clinical trials discussing the safety and efficacy of suzetrigine; however, the results of only six of them were published.

NCT05661734 was a phase III, single-arm study conducted in 256 patients aged 18 to 80 years experiencing moderate-to-severe acute pain. The severity of pain was assessed following surgical procedures or newly developed non-surgical acute pain using the verbal rating scale (VRS) and numeric pain rating scale (NPRS). Participants were administered 100 mg of suzetrigine as an initial dose, followed by a 50 mg dose every 12 h for up to 14 days or until adequate pain relief was achieved. Concomitant use of only ibuprofen (400 mg) and acetaminophen (650 mg) every 6 h was allowed as rescue medication to enhance analgesia in accordance with a multimodal pain management approach. However, opioids and other NSAIDs were not permitted during the treatment period. Suzetrigine demonstrated favorable safety and tolerability, with 83.2% of participants rating their overall response as good, very good, or excellent at the end of treatment. The most frequently reported adverse events were headache, constipation, nausea, fall, and rash. Only 1.6% discontinued treatment with suzetrigine due to insufficient pain relief [119].

NCT04977336 and NCT05034952 were two phase II trials evaluating participants experiencing acute pain after abdominoplasty or bunionectomy. In the abdominoplasty study, 303 participants were randomized into four groups and administrated one of four treatments for two days: (i) a high-dose suzetrigine regimen comprising a 100 mg loading dose (LD) followed by 50 mg every 12 h; (ii) a middle-dose regimen with a 60 mg LD followed by a 30 mg every 12 h; (iii) a control group receiving hydrocodone bitartrate (5 mg) and acetaminophen (325 mg) every 6 h (HB/APAP); and (iv) a group administered a placebo orally every 6 h. In the second trial, 274 bunionectomy participants were randomly divided into five groups receiving oral high-, middle-, or low-dose of suzetrigine for a two-day treatment period. The suzetrigine regimens included a 20 mg LD, followed by 10 mg every 12 h. The remaining groups received either oral hydrocodone bitartrate (5 mg)/acetaminophen (325 mg) or an oral placebo every 6 h. To evaluate efficacy in both trials, the time-weighted sum of the pain-intensity difference over a 48-h period (SPID48) was used to compare suzetrigine with placebo. Analyses from both studies revealed that acute pain was significantly reduced at higher doses, but not at lower doses of suzetrigine over the 48 h. Lack of efficacy led to discontinuation of treatment; however, the percentage appeared to be lower in participants who received a high-dose of suzetrigine compared with the HB/APAP or placebo groups. Suzetrigine did not cause respiratory depression or sedation, whereas headache and constipation were reported more frequently than with placebo in the abdominoplasty trial [1].

In a double-blind, randomized phase III trial (NCT05558410; NAVIGATE 2), patients were enrolled with pain intensity ranging from moderate to severe on the verbal categorical rating system (VRS) and with a score of ≥4 on the numeric pain rating scale (NPRS) within 4 h after undergoing full abdominoplasty. Patients received a 100 mg LD of suzetrigine, followed by 50 mg every 12 h; hydrocodone bitartrate/acetaminophen (HB/APAP) 5 mg/325 mg every 6 h for 48 h; or placebo, in addition to ibuprofen (400 mg) given as a rescue medication every 6 h.

Another randomized, double-blind, phase III trial (NCT05553366; NAVIGATE 1) involved patients who underwent bunionectomy with an NPRS score ≥ 4 during 9 h after the cessation of regional anesthesia following surgery and exhibited the same pain intensity on the VRS. The treatment protocol and assessment criteria, including the use of SPID48, were consistent with those of the previous study. When assessing the time-weighted sum of the pain intensity difference over 48 h (SPID48), a significant reduction in pain was observed in the suzetrigine group (*n* = 447, *n* = 426) in comparison with the placebo group (*n* = 223, *n* = 216), whereas no significant difference was observed between the suzetrigine and HB/APAP groups (*n* = 448, *n* = 431) in either trial. Suzetrigine also demonstrated the shortest time required to achieve pain relief, with 119 min and 240 min compared with 480 min in the placebo group. Twelve percent of participants receiving suzetrigine discontinued the drug due to inadequate efficacy; when compared with discontinuation rates among placebo and HB/APAP recipients, sixteen percent and eight percent were observed, respectively [120].

NCT05660538 was a double-blinded, randomized controlled trial involving patients with moderate to severe painful diabetic peripheral neuropathy and type 1 or type 2 diabetes. Participants were randomly assigned to receive different doses of suzetrigine or pregabalin as a positive control over a 12-week period. Significant reductions in pain were observed with high-dose (69 mg, *n* = 48), mid-dose (46 mg, *n* = 48), or low-dose of suzetrigine (23 mg, *n* = 24) compared with the baseline. These doses produced meaningful changes in the NPRS at week 12, with values of −2.26, −2.11, and −2.18, respectively, whereas the mean change for pregabalin was −2.09. Suzetrigine was generally well tolerated, with adverse events mainly mild or moderate in severity. The most common adverse events were decreased creatinine clearance, dizziness, peripheral edema, and weight increase. One death occurred in the mid-dose group and was attributed to atherosclerotic cardiovascular disease, which was not related to the study medication [59,121].

### 6.2. Registered Clinical Trials

There are many other studies that have been completed but have not yet published their results. Among these are the phase I clinical trial NCT05818852, which evaluated the impact of clinical and high-clinical exposures of suzetrigine and its main metabolite M1 on QTcF, along with the pharmacokinetics, pharmacodynamics, and safety of both [114]. The trial NCT05455502 (phase I) investigated the relative bioavailability of a new suzetrigine tablet formulation and the influence of food in healthy adults [122]. NCT06972212 was another phase I study performed to assess pain tolerance using a cold pressor test [123]. The sensory attributes of the suzetrigine spray-dried dispersion were investigated in the phase I study NCT06834009, in addition to assessing how dose variations influence these sensory attributes [124]. Additionally, a phase II clinical study was performed to evaluate the efficacy and safety of suzetrigine in participants with painful lumbosacral radiculopathy [60].

Other ongoing clinical trials are still recruiting or have not yet started recruitment. The phase IV trial NCT06887972 comprises two single-arm studies evaluating the effectiveness, safety, and tolerability of suzetrigine as part of multimodal therapy in treating acute postoperative pain [125]. Furthermore, the phase IV trial NCT06887959 is assessing suzetrigine for acute pain after laparoscopic procedures of the intraperitoneal or retroperitoneal cavities or arthroscopic orthopedic procedures [126]. The NCT07145346 study evaluates the role of suzetrigine in acute pain relief for patients with multiple rib fractures [127]. The NCT06628908 [128] and NCT06696443 [129] studies are phase III trials investigating the safety profile and efficacy of suzetrigine in participants with painful DPN in the short and long term, respectively.

### 6.3. Limitations

Despite the promising preclinical and clinical evidence supporting the efficacy and safety of suzetrigine as a non-opioid analgesic for the management of acute pain [4], several limitations were identified across the previously reported clinical trials. Notably, a high percentage of female participants, compared with males, were enrolled in phase II and III bunionectomy and abdominoplasty studies. This distribution was representative and reflects the demographic distribution of patients undergoing these procedures in the United States, as reported by Bertoch et al., with approximately 96% and 86% of abdominoplasty and bunionectomy patients being females, respectively [120,130,131]. Furthermore, suzetrigine did not demonstrate gender-based differences in efficacy. However, the existing literature has reported variability between males and females in analgesic responses in chronic pain conditions, whereas no such differences have been observed in acute pain [1,81,120,132,133,134]. Therefore, further studies with a more balanced representation of males and females are needed to evaluate whether there are potential differences in analgesic response between males and females, particularly in cases of chronic pain [81].

Although a significant reduction in acute pain was observed in patients receiving high-dose suzetrigine compared with placebo across two phase II trials (NCT04977336, NCT05034952), both studies used the SPID48 as the primary endpoint. This introduces certain challenges, as SPID48 is a subjective, patient-reported outcome that may not fully reflect well-defined clinical benefits [1].

A notable limitation of the phase II and III abdominoplasty and bunionectomy trials of suzetrigine involves the use of hydrocodone bitartrate (5 mg)/acetaminophen (325 mg) (HB/APAP) as an active comparator administered every 6 h. However, in clinical practice, HB/APAP is administered every 4 h for postoperative pain management. This mismatch in dosing frequency may influence the interpretation of comparative efficacy outcomes [4,81,135].

The selection of an opioid/acetaminophen combination as the active comparator does not perfectly align with Enhanced Recovery After Surgery (ERAS) protocols, which aim to minimize or replace opioid use within multimodal strategies, favoring non-opioid alternatives or acetaminophen as a monotherapy. Therefore, future studies using alternative comparators and clinically optimized dosing regimens are warranted for better assessment of the efficacy of suzetrigine [4].

In a single-arm phase III clinical trial, the efficacy and safety of suzetrigine were evaluated in participants undergoing surgical and non-surgical procedures. However, 86.7% of enrolled participants in the study had postoperative pain, whereas only 13.3% had non-postoperative pain, limiting the ability to draw conclusions regarding the efficacy in non-surgical pain conditions. Moreover, the single-arm study design without an active comparator or placebo control group restricts the interpretation of the outcomes [81,119].

Based on available evidence from clinical trials, suzetrigine demonstrated a favorable tolerability profile, with adverse events of mild-to-moderate severity. The most commonly reported adverse events included headache, constipation, nausea, and rash, with no evidence of respiratory depression, addictive potentials, or central nervous system effects, which are typically associated with opioid therapy. However, the safety follow-up duration across the studies was limited to approximately 14 d, which may be insufficient to adequately assess less frequent or long-term adverse events [1,119,120]. Moreover, a decrease in creatinine clearance was observed in phase II clinical trial in participants with diabetic neuropathy, indicating a potential safety signal that needs further studies, especially in populations with renal impairment [58,121].

## 7. Research Gaps and Future Perspectives

Several ongoing clinical trials are addressing special populations, including patients with renal and hepatic impairment, as these populations may require dose adjustments [115,116]. However, other populations, including geriatric and pediatric patients, as well as those who are pregnant or lactating, need future studies to adequately assess the efficacy and safety of suzetrigine. Additional ongoing trials aim to evaluate potential interactions between suzetrigine and oral contraceptives, in addition to its effects on female fertility [113]. Two further drug–drug interaction studies are investigating possible interactions, especially with CY3P4A inhibitors or inducers, as discussed in the pharmacokinetics section [117,118]. Furthermore, specific cardiovascular safety assessment trials are needed to evaluate adverse cardiovascular events [114]. Phase IV post-marketing surveillance results will be crucial, as they will provide more evidence-based data regarding long-term use, safety, and addictive potential.

Moreover, further studies are needed to evaluate the efficacy and safety of suzetrigine in various chronic pain conditions, including diabetic neuropathy and cancer-associated pain. This represents a significant research gap and highlights the need for a deeper understanding of suzetrigine therapeutic potentials. Additional clinical studies comparing suzetrigine with inhibitors targeting different sodium channel isoforms would provide more robust evidence regarding its selectivity, mechanism of action, and efficacy. Future investigations are also needed to assess the synergistic potential of suzetrigine when used in combination with pharmacological and non-pharmacological therapies, with the aim of maximizing its clinical benefit. Finally, studies evaluating the cost–risk–benefit profile of suzetrigine in comparison with opioids, particularly with long-term usage, are warranted.

## 8. Conclusions

The discovery of suzetrigine represents a significant breakthrough in pain management as it represents the first oral, non-opioid, and selective inhibitor of the Na_V_1.8 sodium channel. It was approved for the treatment of moderate-to-severe acute pain, without serious limitations associated with opioid use, including tolerance and a high risk of addiction. This discovery highlights the therapeutic potentials of targeting Na_V_1.8 sodium channels, which play a crucial role in transmitting pain signals through peripheral pain-sensing neurons. Clinical trials have demonstrated promising results in providing broad-spectrum acute pain relief, especially in surgical pain. Ongoing trials are focused on evaluating its safety and efficacy in additional pain conditions, including postoperative and diabetic neuropathic pain.

Despite the remarkable findings regarding its efficacy, selectivity, and safety profile in acute pain management, further studies are needed to assess the effect of long-term use and its potential in various types of chronic pain. The medicinal-chemical development of suzetrigine may encourage the development of similar drugs. Suzetrigine and future analogues may ultimately pave the way towards a future in which pain can be more safely and effectively managed without reliance on traditional opioid therapy.

## Data Availability

No new data were created or analyzed in this study. Data sharing is not applicable to this article.

## Abbreviations

The following abbreviations are used in this manuscript:

AUC	Area under the curve
CDI	1,1′-Carbonyldiimidazole
CHO	Chinese hamster ovary
C_max_	Maximum plasma concentration
CNS	Central nervous system
CYP450	Cytochrome P450
DCM	Dichloromethane
DIBAL-H	Diisobutylaluminium hydride
DMAP	4-Dimethylaminopyridine
DMF	*N,N*-Dimethylformamide
DPN	Diabetic peripheral neuropathy
FDA	Food and Drug Administration
HB/APAP	Hydrocodone bitartrate/acetaminophen
HEK	Human embryonic kidney
hERG	Human ether-a-go-go-related gene
IPA	Isopropyl alcohol
LD	Loading dose
LSR	Lumbosacral radiculopathy
Na_V_1.8	Voltage-gated sodium channel 1.8
NPRS	Numeric pain rating scale
NSAIDs	Non-steroidal anti-inflammatory drugs
PD	Pore domain
QTcF	QT interval corrected by Fridericia’s formula
SCN10A	Sodium voltage-gated channel alpha subunit 10
SPID	Sum of the pain-intensity difference
TEA	Triethylamine
T_max_	Time to peak concentration
VRS	Verbal categorical rating scale
VSD2	Second voltage-sensing domain
